# Chronic Predation Risk Reduces Escape Speed by Increasing Oxidative Damage: A Deadly Cost of an Adaptive Antipredator Response

**DOI:** 10.1371/journal.pone.0101273

**Published:** 2014-06-26

**Authors:** Lizanne Janssens, Robby Stoks

**Affiliations:** Laboratory of Aquatic Ecology, Evolution and Conservation, University of Leuven, Leuven, Belgium; University of Western Ontario, Canada

## Abstract

Prey organisms evolved a multitude of plastic responses to avoid being eaten by predators. Besides the evolution of plastic morphological responses to escape predation, prey also evolved a set of physiological stress responses to avoid dying because of chronic predator stress *per se* due to disruption of cellular homeostasis. As physiological stress theory predicts increased energy consumption and the inhibition of essential nonemergency body functions, we tested whether chronic predation risk may increase oxidative damage thereby generating negative effects on escape performance. Specifically, we evaluated whether predation risk reduces escape swimming speed in damselfly larvae and whether this operates through stress-associated increases in oxidative damage. Counterintuitively and in contrast with many empirical studies, chronic predation risk decreased escape performance. This is however entirely consistent with the expectation of it being a long-term cost of responding to predation risk (e.g. by increasing respiration or upregulating the stress protein levels). The decreased swimming speed could be explained by an increased oxidative damage to proteins, thereby providing one of the poorly studied ecological links between oxidative damage and whole-animal performance. This likely widespread, understudied cost of chronic predation risk may provide an important pathway of non-consumptive predator effects on prey population dynamics. Moreover, it could play an evolutionary role by acting as a selective force causing prey organisms to adjust the magnitude of the physiological stress response and should be considered when evaluating life history trade-offs thought to be mediated by oxidative damage.

## Introduction

Prey organisms evolved a multitude of plastic responses to avoid being eaten by predators. Considerable attention went to documenting how plastic responses in prey may increase their ability to escape predator attacks. These studies focused almost entirely on predator-induced changes in the prey’s morphology [1]–[3]. Yet, to survive in the presence of predators, prey also need to maintain homeostasis to avoid dying because of chronic predator stress *per se* [4]–[5]; a failure to maintain cellular would indeed ultimately cause death [6]–[7]. Under chronic predator-induced stress, prey therefore evolved a set of adaptive physiological responses such as increased metabolic rates and the allocation of resources to support emergency functions including the upregulation of stress proteins [8]–[10]. Recently it was argued that prey may also improve their escape performance under chronic predation risk as a general consequence of this stress response [11]–[12]. Yet, as these physiological stress responses may alter nutritional budgets and lead to prolonged inhibition of nonemergency body functions and accumulation of destructive effects [6], [10], they may as well reduce escape performance. The impact of chronic predator stress on the prey’s escape performance is directly relevant to understand the selective forces shaping the evolution of the magnitude of the prey’s physiological stress response to chronic predation risk.

One recently discovered destructive effect is that predation risk can result in oxidative damage in prey [13]–[14] which may have wide-reaching negative fitness consequences [15], by increasing metabolic rate and reducing antioxidant defense [16]–[17]. Oxidative damage receives increased attention in ecology as it may provide a mechanistic explanation for costs associated with several types of stressors and may mediate trade-offs between life history and performance [15], [18]–[19]. This negative coupling between oxidative damage and performance can be explained because (i) oxidative damage to lipids can lead to perturbations of membrane structure and function, thereby disturbing signal transduction, and (ii) oxidative damage to proteins can cause damage to the muscle proteins themselves and also impair the functioning of enzymes, signal transduction and transport proteins [20]. Despite the recent burgeoning of interest in how oxidative damage is related to different components of animal performance, this is still poorly documented [15], [21]. A notable indirect observation is the finding that parrots on a diet enriched with antioxidants showed a higher antioxidant capacity and a higher escape flight performance than those on a low quality diet [22]. Together with the recent finding of predator-induced increased oxidative damage [13]–[14], this generates the novel hypothesis that, contrary to common belief, predation risk may reduce a prey’s escape performance by increasing oxidative damage. In line with this hypothesis, it has been suggested that rapid growth may result in a reduced swimming performance through increased oxidative damage to the muscles [23].

We here test if chronic predation risk reduces escape swimming speed through increases in oxidative damage in *Coenagrion puella* damselfly larvae. Damselfly larvae are known to react to predation risk by changing their metabolic rate and energy budget [16], [24] and thereby suffer oxidative damage [14]. Damselfly larvae swim by vigorously moving their abdomen from side-to-side thereby using the three caudal lamellae at the end of the abdomen to generate thrust [25]. Besides effects of predation risk on oxidative damage, we also tested for effects on abdominal muscle mass and lamellae morphology as these variables may also affect swimming speed [26]. Additionally, we evaluated the contribution of all these variables to the escape swimming speed.

## Materials and Methods

### Ethics statement

A collection and rearing permit for damselflies was obtained from “Agentschap voor Natuur en Bos Vlaanderen”.

### Collecting and housing

Penultimate instar larvae of *C. puella* were sampled in a small fishless pond with large dragonfly larvae as top predators in Heverlee (Belgium). In the laboratory, larvae were kept individually in 200 ml cups filled with a mixture of filtered pond water and aerated dechlorinated tap water and fed with *Artemia* nauplii five days a week (average daily dose = 604, SE = 36, n = 10). The cups were placed in an incubator at 22°C and a 14∶10 L:D photoperiod. When larvae molted into the final instar (>15 days after collection), they were used for the experimental trials. Starting from that point, larvae were fed daily.

### Experimental design

Larvae were exposed to one of two predation risk treatments (absence versus presence) for seven days. A 7-day exposure period can be considered to impose chronic predation risk [27]. In previous studies on damselfly larvae, effects of predation risk on physiology were detectable after exposure periods of 4 days [28]–[30]. During the exposure period, larvae were placed individually in glass vials (100 ml) filled with 50 ml water with daily replacement of the medium. Glass vials were placed in groups of four in larger containers (750 ml) and daily randomly re-distributed among containers of the same treatment. Predation risk was manipulated using a combination of visual and chemical predator cues, reflecting the cocktail of predator cues damselfly larvae encounter in nature.

To provide visual predator cues, a large field-collected *Anax imperator* dragonfly larva was placed in the containers of the treatment with predation risk. Additionally, *C. puella* larvae could see the conspecific larvae in the other vials in the container (damselfly larvae are cannibalistic [31]). To preclude visual predator cues in the condition without predation risk, the sides of the vials of this condition were made non-transparent using tape. Note that as vials were illuminated from above, this did not affect the light intensity in the vials. For the chemical predator cues, we daily homogenized one *C. puella* larva in 20 ml of water from an aquarium filled with 300 ml aged tap water in which a large *Anax* dragonfly larva had eaten a *C. puella* larva. Every 24 h (based on [32]), when replacing the medium, we added 1 ml of this predator medium to each vial of the predation risk treatment. To the vials of the treatment without predation risk we added 1 ml of aged tap water.

### Response variables

The experiment was run twice. In a first run, we quantified effects of predation risk on final swimming speed using 30 larvae per treatment; on a subset of 10 larvae per treatment we also quantified oxidative damage to lipids and to proteins and abdominal muscle mass. In a second run, to get a more complete picture and rule out confounding effects of initial swimming speed and effects on lamellae morphology, we measured more variables. Here, we measured swimming speed at the start and at the end of the exposure period, oxidative damage to lipids and to proteins and the amount of muscle mass, and lamellae morphology on 20 larvae per treatment.

At the beginning and at the end of the 7-day exposure period we measured the escape swimming speed. Damselfly larvae use swimming to escape predation by dragonfly larvae [26], [33]–[34]. At the start of a swimming trial, we transferred a single larva to a container (20 cm×12 cm×8 cm), filled with 1 l of aged tap water. The larva was stimulated to swim by tapping it on the dorsal surface of the thorax with a plastic pipette and three swimming bouts per larva were filmed using a high speed camera (Basler pi A 640, 200 Hz) connected to a computer using Streampix software. From these recordings we quantified per swimming bout the swimming speed using Image Pro Plus v5. Swimming speed (cm/s) was calculated as the distance the larvae covered during the first 100 frames of one swimming bout divided by the duration (0.5 s). We chose to digitize the first 0.5 s to have enough frames to accurately calculate swimming speed while this initial 0.5 s period is likely to be the most critical period for damselfly larvae to escape attacks from sit-and-wait predators such as dragonfly larvae that do not chase their prey after the initial attack [35]. Per larva, we used the fastest of the three swimming bouts for later analysis.

In order to test for effects of predation risk on larval mass and effects of mass on swimming speed, we weighed each larva to the nearest 0.01 mg using a microbalance before the exposure period and at the end of the exposure period (when we did the swimming test). There was no difference in body mass between the two treatments neither in the pre-exposure period (t_98_ = −0.52, p = 0.61; control: 23.84±0.52 mg, predation risk: 23.46±0.52 mg), nor at the end of the exposure period (t_98_ = −1.39, p = 0.17; control: 27.42±0.60 mg, predation risk: 26.34±0.60 mg).

On a subset of 20 larvae per treatment we quantified lamellae morphology [25]. We digitized each lamella with a microscope (Olympus BX50, magnification 10×) attached to a camera (Olympus DP50) using the computer program Olympus DP50 Soft and quantified the area, length and width using Cell P Plus.

We measured oxidative damage and muscle mass on 30 larvae per treatment. The abdomen was 15 times diluted, homogenized in phosphate buffer (PBS, 50 mM, pH 7.4) using a pestle and centrifuged (16,100 g, 4°C). The resulting supernatant was used for the physiological analyses. Note that by measuring oxidative damage in the abdomen supernatant we likely measured oxidative damage to muscle tissue.

As swimming muscles of damselfly larvae make up most part of the abdomen (Lizanne Janssens, unpublished data), we estimated the amount of swimming muscles by quantifying the protein content of the abdomen [36]. For this, we used the protocol of Bradford [37]. We mixed 1 µl supernatant, 160 µl mili-Q water and 40 µl Bio-Rad Protein Dye in a 96 well plate. After an incubation of 5 minutes at 30°C, we measured absorbance at 595 nm. We estimated the swimming muscle mass as the protein content of the abdomen based on a standard curve of known concentrations of bovine serum albumin and expressed it in µg/mg abdominal wet mass.

Fat content was quantified based on the protocol of Bligh and Dyer [38]. We filled a 2 ml glass bottle with 8 µl of the supernatant and 56 µl sulfuric acid (100%). The bottles were heated for 20 minutes at 150°C. Afterwards 64 µl mili-Q water was added. We filled a transparent 384 well microtiter plate with 30 µl of the sample and measured absorbance at 340 nm. Fat concentrations were calculated using a standard curve of glyceryl tripalmitate.

To measure oxidative damage to proteins, we quantified one of the most often used biomarkers for this, the level of carbonyls [15]. Carbonyls are introduced into proteins by direct oxidation of amino acids or indirectly by attachment of a carbonyl-containing moiety. The carbonyl content was quantified using the OxiSelect Protein Carbonyl ELISA kit STA-310 of Cell Biolabs Inc. In a first step, the supernatant was diluted to obtain a protein concentration of 10 µg/ml. Afterwards, 100 µl of the diluted supernatant was added to a 96 well protein binding plate. After an overnight incubation at 4°C, the wells were washed three times with 250 µl PBS. Next, 100 µl DNPH working solution was added. After an incubation of 45 minutes at room temperature in the dark, the wells were washed five times with 250 µl PBS/ethanol (1∶1 v/v) with 5 minutes incubation and two times with 250 µl PBS. From this point on, each incubation was done on an orbital shaker. Afterwards, 200 µl blocking solution was added and the samples were incubated for 1.5 h at room temperature. After washing the samples three times with 250 µl wash buffer, 100 µl diluted anti-DNP antibody (dilution 1∶1,000) was added and the mixture was incubated at room temperature for 1 hour. Then, the samples were again washed three times with 250 µl wash buffer and 100 µl diluted HRP conjugated secondary antibody (dilution 1∶1,500) was added. After an incubation of 1 hour at room temperature, the samples were washed 5 times with 250 µl wash buffer and 100 µl substrate solution was added. The mixture was incubated for 20 minutes at room temperature. The enzymatic reaction was then stopped by adding 100 µl Stop Solution and absorbance was measured at 450 nm. The carbonyl concentrations were calculated based on a standard curve of known concentrations of reduced and oxidized BSA and expressed as nmol carbonyls/mg protein.

We measured oxidative damage to lipids by measuring an often used biomarker of lipid peroxidation, the formation of malondialdehyde (MDA) [15]. Sample preparation was based on the protocol described in Miyamoto et al. [39]. First, 50 µl supernatant and 50 µl TBA 0.4% were mixed (40 mg TBA in 10 ml 0.2 M HCl). This mixture was incubated at 90°C for 60 minutes and cooled on ice. Afterwards, we added 165 µl n-butanol, mixed and centrifuged the mixture for 3 minutes (4°C, 16,100 g). Finally, 10 µl of the butanol fraction was injected in an HPLC/UV-Vis system on a C18 column (250×4.6×5 µm) (protocol by Karatas et al. [40]). The mobile phase was 30 mM KH_2_PO_4_-methanol (65+35, v/v %, pH 4); the flow rate was isocratic, 1 ml/min. Chromatograms were monitored at 535 nm and the retention time of MDA was 3.88 min. A standard curve was established using 1,1,3,3-tetraethoxypropane (TEP, malonaldehyde, bisdiethylacetal). MDA concentrations were expressed in nmol MDA/mg fat.

### Statistical analyses

We performed separate ANOVAs to test for effects of predation risk and experimental run (first vs second run of the experiment) on the different response variables. Significant interactions were further explored using Duncan post hoc tests. MDA levels were log-transformed in order to meet the assumptions. We initially included body mass as a covariate for swimming speed, but since it was not significant (p = 0.20), it was removed from the model.

To explore the contribution of oxidative damage to changes in swimming speed, we used an ANCOVA with predation risk as categorical variable, carbonyl and MDA levels as continuous covariates and swimming speed as the response variable. Similarly, we tested for covariation patterns between abdominal muscle mass, lamellae morphology and swimming speed. We initially included the interactions with the covariates and the predation risk in the model, but as these were never significant (all p>0.17) they were removed from the final model. There were no correlations between the abdominal muscle mass and the levels of oxidative damage (all p>17). All tests were done in STATISTICA 11.

## Results

**Figure 1:**
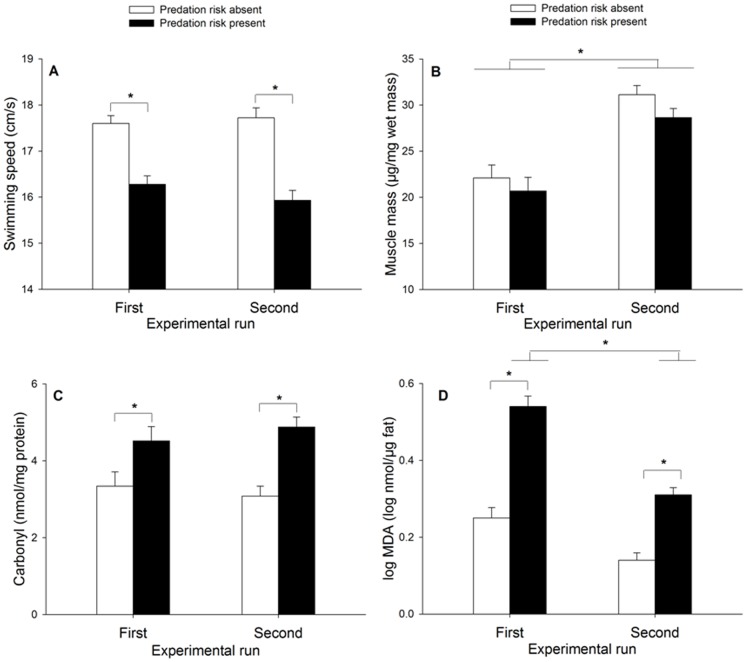
Effect of predation risk on swimming speed, abdominal muscle mass and oxidative damage. Mean (+1 SE) swimming speed (A), abdominal muscle mass (B), oxidative damage to proteins (carbonyl levels) (C) and oxidative damage to lipids (MDA levels) (D) of *C. puella* damselfly larvae as a function of experimental run and exposure to predation risk.

There was no difference in initial swimming speed between larvae of both predation risk treatments (F_1, 38_ = 0.60, p = 0.44; control: 19.13±0.29 cm/s, predation risk: 18.81±0.29 cm/s). Damselfly larvae exposed to predation risk had a lower escape swimming speed at the end of the 7-day exposure period (F_1, 96_ = 60.06, p<0.001; Figure 1A). This effect of chronic predation risk on escape speed was consistent across experimental runs (predation risk×experimental run, F_1, 96_ = 1.41, p = 0.24; Figure 1A). There was no effect of the experimental run on swimming speed (F_1, 96_ = 0.32, p = 0.57).

There was no effect of predation risk on the abdominal muscle mass (F_1, 55_ = 1.24, p = 0.27), and this was consistent across experimental runs (predation risk×experimental run: F_1, 55_ = 0.01, p = 0.91). Larvae tested in the second experimental run had a higher abdominal muscle mass (F_1, 55_ = 39.91, p<0.001; Figure 1B). Predation risk did not affect the lamellae morphology (area: F_1, 37_ = 0.43, p = 0.51; control: 1.63±0.19 mm^2^, predation risk: 1.45±0.19 mm^2^; length: F_1, 37_ = 0.79, p = 0.38; control: 2.89±0.20 mm, predation risk: 2.63±0.21 mm; width: F_1, 37_ = 0.07, p = 0.80; control: 0.63±0.05 mm, predation risk: 0.61±0.05 mm).

Both measures of oxidative damage increased under predation risk (carbonyl: F_1, 56_ = 5.18, p = 0.03; MDA: F_1, 56_ = 98.31, p<0.001; Figure 1C–D). This increase was consistent across experimental runs for the carbonyl levels (experimental run: F_1, 36_ = 0.76, p = 0.39; predation risk×experimental run: F_1, 36_ = 0.0002, p = 0.99). MDA levels were higher in the first experimental run (F_1, 56_ = 52.74, p<0.001) and the increase in oxidative damage under predation risk was also stronger in the first run (predation risk×experimental run: F_1, 56_ = 5.37, p = 0.02), yet the difference between the two treatments was significant in both runs (Duncan post hoc tests, first run: p<0.001; second run: p<0.001).

**Figure 2:**
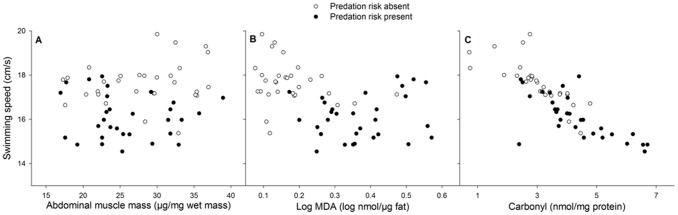
Covariation patterns between swimming speed and abdominal muscle mass and oxidative damage. Covariation patterns between swimming speed and (A) abdominal muscle mass, (B) oxidative damage to proteins and (C) oxidative damage to lipids in *C. puella* damselfly larvae.

Carbonyl levels covaried negatively with swimming speed (F_1, 54_ = 99.36, p<0.001; slope ±1SE = −0.67±0.07; Figure 2C). MDA levels and the abdominal muscle mass did not covary with swimming speed (MDA: F_1, 54_ = 1.97, p = 0.17; slope ±1SE = −0.16±0.12; muscle mass: F_1, 54_ = 1.05, p = 0.31; slope ±1SE = 0.003±0.003) (Figure 2A–B). Lamellae morphology did not covary with swimming speed (size: F_1, 34_ = 0.67, p = 0.42; slope ±1SE = −0.0002±0.0002; length: F_1, 34_ = 0.13, p = 0.72; slope ±1SE = 0.004±0.010; width: F_1, 34_ = 1.02, p = 0.32; slope ±1SE = 0.05±0.05).

## Discussion

The key novel finding of our study was that, contrary to general belief, the escape speed was reduced under chronic predation risk. This was not caused by plasticity in the abdominal muscle mass or lamellae morphology (this study), nor by any changes in food intake (Janssens & Stoks, unpublished data). A general stress-related mechanism associated with chronic predation risk could be explicitly linked to the reduction in escape speed: an increase in oxidative damage to proteins. Our findings have important fitness consequences as damselfly larvae strongly rely on escape swimming when attacked by predators [28] and predators such as dragonfly larvae impose positive survival selection on escape swimming speed [26], [34].

Prey evolved a set of adaptive physiological responses to prepare them to escape predation and to maintain physiological homeostasis under chronic predation risk. Such responses have been well documented in damselfly larvae and include an increase in respiration and the upregulation of stress proteins [16]. These protective responses are crucial to allow animals to deal with chronic predation risk which can reach lethal levels [4]–[5]. However, they also alter nutritional budgets and cause prolonged inhibition of essential nonemergency functions which may result in the accumulation of destructive effects [6], [9]–[10]. One such inhibited nonemergency body function that is reduced under chronic predation risk is antioxidant defense [14], [16]. Together with the typically increased respiration under predation risk [16], [41], this is expected to impose oxidative damage. We here confirm the only two other studies demonstrating an increase in oxidative damage under predation risk [13]–[14]. We hypothesize that such predator-induced increases of oxidative damage are widespread in prey given the generality of the combination of the two above-mentioned mechanisms. More importantly, we could demonstrate that the predator-induced increase in oxidative damage contributed to the escape speed reduction. This negative coupling between oxidative damage and swimming speed can be explained as damage to the muscle proteins themselves or the impairment of the functioning of enzymes, signal transduction and transport proteins [20].

Our findings make important contributions to several research topics in predator-prey ecology, oxidative-stress ecology and general stress ecology that are increasingly gaining attention. First, there is a surge of interest in non-consumptive effects of predators as these may be as important as consumptive effects in shaping prey population dynamics [42]. Especially non-consumptive effects on physiology have been understudied [43]. Given the likely consequences for consumptive effects, the here documented stress-induced reduction in escape performance has the potential to provide a novel pathway of non-consumptive predator effects on prey population dynamics. Second, our study provides the first direct evidence for a link between oxidative damage and escape performance thereby providing one of the poorly studied links between oxidative damage and whole-animal performance [15], [18]. This complements the observation that parrots kept on a diet enriched with antioxidants showed a higher antioxidant capacity and a higher escape performance than those on a low quality diet [22]. The demonstration of the negative coupling between oxidative damage and swimming speed provides an important mechanistic proof-of-principle that is relevant to understand other key trade-offs in ecology.

Our results also contribute to the understanding of the evolution of the magnitude of the physiological response to avoid predation by documenting one important cost in terms of reduced escape performance. While the predator-induced physiological stress response constitutes an adaptive set of physiological responses that evolved to avoid predation, the increased metabolic rate and shunting of energy away from other functions may also generate costly physiological effects such as the increase in oxidative damage that we have documented here. While it may seem counterintuitive that as a result prey develop lower escape speeds under chronic predation risk, we hypothesize that the need to maintain homeostasis and avoid dying because of chronic predator stress *per se* [4]–[5] outweighs the increased risk of being killed by dragonfly predators given the reduced escape speed. In other words, this may be a long-term fitness cost of a set of evolved adaptive responses that promote fitness [10]. The here documented cost in terms of a reduced escape performance could play an evolutionary role by acting as a selective force causing prey organisms to adjust the magnitude of the physiological stress response to the perceived levels of risk and by reducing the strength of the physiological stress responses over time [6].

Oxidative damage is increasingly considered as an important mediator of life history trade-offs, thereby potentially acting as a selective force shaping life history evolution [17], [44]–[45]. Few studies, however, explicitly identified how it can impair performance. The here identified link between reduced escape speed and increased oxidative damage to proteins may be an important, yet overlooked, cost and should therefore be considered when evaluating life history trade-offs thought to be mediated by oxidative damage.
